# Provision of Rational Parameters for the Turning Mode of Small-Sized Parts Made of the 29 NK Alloy and Beryllium Bronze for Subsequent Thermal Pulse Deburring

**DOI:** 10.3390/ma16093490

**Published:** 2023-04-30

**Authors:** Nikita V. Martyushev, Dmitriy A. Bublik, Vladislav V. Kukartsev, Vadim S. Tynchenko, Roman V. Klyuev, Yadviga A. Tynchenko, Yuliya I. Karlina

**Affiliations:** 1 Department of Advanced Technologies, Tomsk Polytechnic University, 634050 Tomsk, Russia; 2 Department of Thermal Power Engineering, Institute of Information Technologies and Data Analysis, Irkutsk National Research Technical University, 664074 Irkutsk, Russia; 3 Department of Informatics, Institute of Space and Information Technologies, Siberian Federal University, 660041 Krasnoyarsk, Russia; 4 Department of Information Economic Systems, Institute of Engineering and Economics, Reshetnev Siberian State University of Science and Technology, 660037 Krasnoyarsk, Russia; 5 Digital Material Science: New Materials and Technologies, Bauman Moscow State Technical University, 105005 Moscow, Russia; 6 Department of Technological Machines and Equipment of Oil and Gas Complex, School of Petroleum and Natural Gas Engineering, Siberian Federal University, 660041 Krasnoyarsk, Russia; 7 Information-Control Systems Department, Institute of Computer Science and Telecommunications, Reshetnev Siberian State University of Science and Technology, 660037 Krasnoyarsk, Russia; 8 Technique and Technology of Mining and Oil and Gas Production Department, Moscow Polytechnic University, 33 B. Semenovskaya Str., 107023 Moscow, Russia; 9 Laboratory of Biofuel Compositions, Siberian Federal University, 660041 Krasnoyarsk, Russia; 10 Department of Systems Analysis and Operations Research, Reshetnev Siberian State University of Science and Technology, 660037 Krasnoyarsk, Russia; 11 Stroytest Research and Testing Center, Moscow State University of Civil Engineering, 26 Yaroslavskoe Highway, 129337 Moscow, Russia

**Keywords:** edge processing, thermal impulse processing, processing of beryllium bronze, processing of alloy 29 NK, deburring

## Abstract

The increase in the share of physical and technical processing methods in the arsenal of deburring technologies used in modern production is associated both with the use of difficult-to-machine materials, such as beryllium bronze and the 29 NK alloy, and with the need to solve technological problems for the production of small-sized products with hard-to-reach surfaces. The aim of the study is to improve the processes of blade processing of small-sized parts made of beryllium bronze and the 29 NK alloy to provide rational conditions for thermal pulse deburring. Surface samples were experimentally obtained after turning in different modes on a CITIZEN CINCOM K16E-VII automatic lathe equipped with an Applitec micromechanics tool. The surface quality and burr characteristics were examined using a JEOL JIB-Z4500 electron microscope and a ContourGT-K optical profilometer. The program Statistica 6 allowed processing of the results. The relationship between the parameters of the turning mode and the thickness of the root of the burrs formed on the machined surface, the limitation of which is one of the conditions for the application of the thermal pulse method, was established. The obtained empirical regression dependencies establish a rational range of cutting mode parameters, and the implementation of the formulated recommendations for setting blade modes ensures deburring by the thermal pulse method in compliance with the requirements of drawing under maximum processing performance.

## 1. Introduction

The modern development of equipment and technologies in the field of metalworking has led to an increase in the range of small-sized, high-precision parts made of difficult-to-machine materials, the processing of which is carried out on CNC machines in such industries as instrument making and radio-electronics. A significant part of the electronic component base is made up of coaxial radio components, the conductors of which are made of beryllium bronze and the 29 NK alloy [1]. Based on the analyzed labor intensity of manufacturing small-sized high-precision parts made of beryllium bronze and the 29 NK alloy using CNC machines, one may conclude that a significant proportion of labor costs are associated with operations aimed at removing burrs from parts on which high requirements for dimensional accuracy and surface roughness are imposed.

The scientific problem is that the parts under study are small (the body of revolution of a diameter is from 0.4 to 10 mm, up to 15 mm long), have high accuracy (of 7–10 quality), entail a complex geometry of internal hard-to-reach surfaces of external conductors, and include thin-walled construction elements. Burrs and sharp edges are not allowed [2,3]. It is not possible to exclude the formation of burrs at the cutting stage. The presence of internal hard-to-reach surfaces complicates the process of deburring by mechanical methods. Burrs are removed from internal surfaces by manual metalwork.

In practice, deburring is realized by mechanical methods. The geometric features of parts affect the complications of deburring. If there are no internal surfaces, tumbling is used (grinding and polishing in two or three solutions with different fillers for 0.5–1 h). If there are internal surfaces and the external surfaces are tumbling, then manual deburring on internal hard-to-reach surfaces is carried out. This deburring method is characterized by low productivity and variable quality.

When processing by mechanical means, abrasive grains that can penetrate hard-to-reach surfaces are small and damage the surface; processing is carried out using large granules that act on elements, which protrude above the common surface (burrs). Complete deburring cannot be achieved by installing steel or ceramic brushes on the machine [4]. The rubber-bonded abrasive wheels installed on the machine are destroyed by the oil cutting fluid.

Methods for removing burrs according to the method of exposure are divided into two groups. The mechanical methods include manual, semi-automated, and automated processing with a special abrasive cutting brush tool; processing occurs in an environment of free abrasives with various methods of delivering the abrasive grains to the treated surfaces. The physical and technical methods involve ultrasound, electrohydropulse processing, thermal energy, thermal pulse, electrochemical, and electrocontact methods [5].

Promising methods for removing burrs without using manual processing include physical and technical methods designed to remove burrs from internal hard-to-reach surfaces without damaging them.

The thermal pulse method has important advantages; it is stable, reliable, and enables complete removal of all burrs from all surfaces of small parts, with low time costs. The limitation of the use of the thermal pulse method is the size of the removed burrs, the size of which is not more than 0.1 mm for workpieces made of beryllium bronze, and not more than 0.3 mm for workpieces made of the 29 NK alloy. However, the size must not be more than 1/4–1/6 of the minimum thickness of the workpiece. Securing parts requires special equipment.

The thermal-pulse method is designed to remove non-liquids and burrs from parts of a complex profile where only gas can penetrate. The main parameters influencing treatment by the thermal-pulse method are the gas–oxygen mixture composition, the combustion temperature, and the volume and pressure of the gas–oxygen mixture. The gas–oxygen mixture composition is regulated by the stoichiometric ratio for mixing the gases. Oxygen performs two tasks. It is necessary to burn the fuel gas because the fuel gas must react with oxygen and, thereby, consume it to release heat. One portion of propane and two portions of oxygen are burnt, which is accompanied by the formation of carbon dioxide and water. This results in the fact that, if the mixing ratio of C3H8:O2 = 1:2, the entire fuel gas reacts with oxygen; a little more oxygen is added to oxidize burrs. At the same time that burning takes place, the temperature reaches its maximum. When an excessive amount of oxygen is injected into the deburring chamber, oxygen can perform its second task—burr burning. The more oxygen that is available, the greater the proportion of the material that is removed. If the oxygen content is too high, there will be no deburring because the burrs cannot be heated to the flame temperature. The gases are dosed through the gas metering system present in the plant intended for thermal deburring of the parts. When processing in a rationally selected mode, the surface roughness is slightly reduced; the burrs are completely removed but the geometry of the part does not change. The part can be prepared for subsequent thermal pulse processing in various ways. It is possible to either remove or reduce the size of the burrs by means of barrel polishing. At the same time, internal surfaces are not processed. Barrel polishing also influences the surface of the product (polishing, grinding, edge rounding). A rubber-based brush treatment can be used as a pretreatment. Such brushes fail very quickly, and such treatment may cause difficulties when complex and internal surfaces are processed. Using ceramic brushes does not require their frequent replacement, but such brushes may damage the product surface. Manually removing large burrs using a microscope can solve the problem. However, this is an extremely laborious, time-consuming, and expensive operation.

To solve this problem, a two-stage process is proposed. Minimizing the size of burrs at the cutting stage will provide the conditions for high-performance thermal pulse deburring.

Based on literature review, the works of S. Bratan et al. [6], V. Bobrov [7], V. Bezyazyachny et al. [8], and O. Erenkov et al. [9] were found to be devoted to the stated problem. The works of B. Mokritsky, et al. [10] considered the problems of increasing the efficiency of cutting on CNC machines. These issues were dealt with by B. Ponomarev et al. [11], V. Davydov et al. [12] and L. Shan et al. [13] who describe the main methods and tools for measuring and controlling holes in terms of the accuracy of the diametrical size, deviations from roundness and cylindricity, and the relative position of the axis. A. Richter [14] described how to safely and effectively machine beryllium. O. Koichi et al. [15] studied the surface integrity of beryllium copper alloy by microcutting. S. Prasad [16] performed a comprehensive study of the wire electrical discharge machining of beryllium-copper alloys.

A. Shokrani [17] presented the first research on investigating the machinability of the Kovar, Fe-29Ni-17Co alloy, which is used in the electronics and optoelectronics industries. The cutting tool geometry and machining environments were investigated as major factors. H. Wasynczuk et al. [18] studied the high-cycle fatigue of Kovar. However, these studies do not describe the effect of the cutting conditions on the characteristics of the resulting burrs. The issue of cutting small-sized workpieces made of beryllium bronze and the 29 NK alloy has not been sufficiently studied. The ability to control the burr size by setting rational cutting parameters will improve deburring performance.

The purpose of this study is to improve the processes for the blade processing of small-sized, high-precision parts made of beryllium bronze and the 29 NK alloy to provide rational conditions for subsequent physical and technical processing by thermal pulse deburring.

The research objectives are as follows:

(a) to evaluate the influence of the parameters of blade processing of small-sized, high-precision parts made of beryllium bronze and the 29 NK alloy on the performance of the deburring process;

(b) to conduct a set of experimental studies on the effect of the blade processing modes of small-sized, high-precision parts made of beryllium bronze and the 29 NK alloy on the parameters of burrs;

(c) to establish empirical regression dependencies that make it possible to describe the effect of cutting modes of small-sized, high-precision parts made of beryllium bronze and the 29 NK alloy on the burr parameters;

(d) to develop recommendations for setting rational parameters for the blade processing of small-sized, high-precision parts made of beryllium bronze and the 29 NK alloy.

The object of the study is the blade processing of workpieces of coaxial radio components made of beryllium bronze and the 29 NK alloy of a diameter within 0.4–10 mm, a length of 4–15 mm, and an accuracy of 7 quality.

The subject of the study is the dependence of burr parameters on the parameters of the turning mode of workpieces made of beryllium bronze and the 29 NK alloy, as well as the relationship between the burr size and deburring performance.

## 2. Materials and Methods

### 2.1. Materials Employed

Workpieces made of beryllium bronze Rod BrB2 of 5 mm in diameter (ASTM B197) [19] and 29 NK precision alloy, having a given temperature coefficient of a linear circle diameter of 6 mm (29 NK (Kovar) alloy, ASTM F15-04) [20] were studied. The material properties of the beryllium bronze and 29 NK alloy are shown in Table 1 and Table 2, respectively.

The chemical compositions of beryllium bronze UNS C17300 and 29 NK alloy are outlined in the following Table 3 and Table 4.

### 2.2. Sample Preparation

These materials are difficult to cut because of their physical and chemical properties. Experiments were conducted in the form of full-scale experiments; this involved experimental selection of cutting modes and tools, in which the thickness of the burr root did not exceed the established norm. Workpieces for studying the dependence of the thickness of the burr root on the parameters of the turning mode were obtained on a CITIZEN CINCOM K16E-VII longitudinal turning lathe designed for micromechanics, using an Applitec carbide, with cutting tool-inserts for using the holder system 740-12-90. To machine the beryllium bronze samples, the 744-1.0-R cross-turning insert, 1.0 mm in size, employed an easily regrindable rake geometry recommended by the manufacturer as the initial choice for the parts made of brass and bronze. This was also recommended when handling brittle or small copper parts and specimens made from the 29 NK alloy. The manufacturer recommends using inserts for the 744 ×-1.5-R transverse turning of a size of 1.5 mm, having a standard positive geometry of the front surface, as the initial choice for parts made of steels and hard alloys. The 740-12-90 insert holder was used for fastening the inserts, which involved a patented Applitec rigid fastening system and an A-B type of fastening, in which the insert could be changed without removing the holder from the machine. Turning was carried out using an oil coolant medium of medium viscosity. Measuring the thickness of the burr root having a maximum value of the root thickness on the sample was carried out using a workpiece made of the Rod BrB2 material. This was a drawn round rod of increased manufacturing accuracy; it was hard (after hardening), 5.0 mm in diameter and of a random length. Another one was made from bronze of the BrB2 brand. The same process was performed with a workpiece made from the Circle 29 NK material. This was a bar having a diameter of 6.0, in group B of quality h7, made from the 29 NK alloy after turning to L = 5 mm length.

### 2.3. Analytical Studies

Analytical studies of the burr parameters were carried out according to the provisions of the theory of metal cutting, mathematical statistics and the design of experimental methods, and analysis of factors affecting the degree of burr removal during thermal pulse surface treatment of parts. The characteristics of burrs were studied by means of a microscope.

### 2.4. Determination of Burr and Surface Roughness Parameters

Burr characteristics and dimensions were analyzed using an SEM Multi-Beam System JEOL JIB-Z4500 scanning electron microscope (Tokyo, Japan). The roughness was measured in a non-contact way using an optical profilometer ContourGT-K [21].

## 3. Results

### 3.1. Influence of Blade Processing Parameters of Small-Sized, High-Precision Parts Made of Beryllium Bronze and the 29 NK Alloy on Deburring Process Productivity

The performance of a deburring process depends on the deburring method used. The limitation of using deburring methods is the characteristics of the products, in terms of:

the geometric dimensions;

the accuracy parameters;

the requirements for surface quality;

the physical and mechanical properties of the used materials;

the number, size and location of burrs.

For the workpieces of the parts of the coaxial radio components made of the BrB2 beryllium bronze and 29 NK alloy, small geometric dimensions, high requirements for accuracy and surface quality, the presence of internal hard-to-reach surfaces, and thin-walled and easily damaged structural elements are typical. The used material, beryllium bronze BrB2, has high elasticity [19], which contributes to the formation of long drain chips during blade processing, and, as a result, the formation of firmly attached burrs over the entire surface of the workpiece. The 29 NK alloy is characterized by high hardness, which contributes to the formation of strong, hard-to-remove burrs on the surface of the workpiece. The 29 NK alloy contains 29% nickel, 17% cobalt, and 54% iron; the Brinell hardness is HB 10^−1^ = 161 MPa, and the Vickers hardness is HV = 160 per 1 kgF [20]. The number and size of burrs increases by 1.5–2 times as the tool blade wears out. High accuracy parameters (up to 7 quality), and the presence of critical, easily damaged structural elements, such as threads, make it impossible to use deburring methods that damage the surface.

The main parameters of burrs that affect the method and time of their removal are:

the dimensions (height, thickness, length);

the hardness;

the cross-sectional shape;

the configuration in the longitudinal direction;

the location (accessibility) [4,22].

The root of the burr is the part adjacent to the surface to be machined. The top of the burr is its end, which is the burr thickness away from the surface to be machined. The thickness of the burr is the thickness of the burr root, determined in its cross-section along the machined surface. The height of the burr is the size determined perpendicularly to the machined surface starting from the root to the part of the burr furthest from the surface [4]. The length of the burr is the size that characterizes the extent of the burr along the machined surface. The hardness of the burr is the hardness of the burr root, measured on the theoretical line of the machined surface [4].

**Figure 1:**
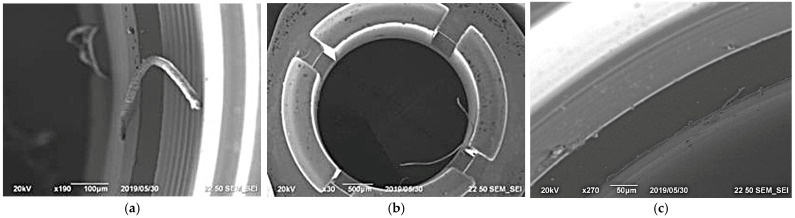
Burrs obtained in different modes of processing blanks made from the BrB2 beryllium bronze BrB2: (**a**) long stretching burrs having several peaks and one root; (**b**) burrs that are strongly attached to the part surface in the areas located along the burr length; (**c**) processing based on experimentally selected parameters at n = 6000 rpm, S = 0.03 mm/rev.

The cross-section of burrs on the parts made of beryllium bronze, brass and the 29 NK alloy, formed during cutting, has a shape close to round; the thickness of the burr root reaches 0.05 mm. They have a reinforced base, being evenly strong along the entire length, and it is difficult to break them off [4]. These are long, trailing burrs, often consisting of a single root and several vertices. The burrs obtained in the modes that do not provide the required heat removal have areas of attachment to the surface of the part in separate sections located along the length of the burr; in this case, the root of the burr has the shape of a long oval—the root thickness increases significantly and reaches 0.1 mm. They stretch, and welding them to the surface of the part requires time. The appearance of such burrs having a large attachment area to the surface of the parts is critical for choosing further deburring methods. Figure 1 shows the burrs that are characteristic of various processing modes.

The burrs shown in Figure 1a were obtained in the processing modes recommended by the manufacturer using an unworn tool. There were many such burrs, and they were difficult to remove manually; when using a scraper, there was a high probability of cutting into the depth of the surface of the part. The burrs shown in Figure 1b were obtained at feed and cutting speeds closer to the lower limit of the recommended range using a tool with a high degree of wear. The burrs shown in Figure 1b were firmly attached to the surface of the part and were very difficult to remove manually; the area of their attachment to the surface of the part could exceed the standard thickness of the burr root intended for removal by the thermal pulse method. By reducing the feed and cutting speed and setting the depth of the cut, the creation of chips that were suitable for heat removal and did not clog the tool could be ensured. In the process, the burrs were shortened, become tear-shaped, and the root thickness decreased. Figure 1c shows the results of processing based on the experimentally selected parameters with the number of spindle revolutions of n = 6000 rpm, a 5-mm diameter, and a feed of S = 0.03 mm/rev on a new tool. The size and number of burrs having these processing parameters were minimal.

Depending on the geometry, the parts can be divided into two groups: parts that do not have internal surfaces and those that have internal hard-to-reach surfaces.

The parts that did not have internal surfaces were subject to abrasive processing. On the outer surfaces of the parts that were not subject to abrasive processing, for example, those containing threads, protective covers needed to be applied. The abrasive machining of the parts was tested in a MultiFinish MF-5 centrifugal finishing machine (MultiFinish GmbH & Co. KG, Baden-Wurttemberg, Germany), but it was not possible to select modes in which the surface of the small parts remained undisturbed and all burrs were removed. Processing by such a machine was used for rougher processing, and the deburring of larger parts. In the case of the abrasive cleaning of parts of coaxial radio components, the Kyngty jewelry machine tumbling drum was most suited, providing a more delicate treatment. The quality of abrasive processing also depended on the used fillers. The best result was obtained by tumbling in an aqueous solution of Super COMPOUND 3–4% SC 36 OTEC supplemented with fillers used for wet grinding (coarse processing) and white ceramic prisms GA—10 × 10 (medium aggressiveness) or DZS 4 × 4 OTEC for 0.5–1 h at speed 3. The same process was performed with the wet polishing compound (fine finish) having ceramic prisms of green OTEC PM-10 for 0.5–1 h at speed 3. Then, the wet polishing compound (finishing, essentially fine polishing due to the round shape filler) was followed using GA granules of a 4-mm diameter round porcelain for 0.5 h at speed 2 or 3. These fillers were of fairly large size and had low aggressiveness, and gradually smoothed the surface, grinding off burrs, but without penetrating the surface between them or breaking it. The duration of the first and second stages of the abrasive processing in the tumbling drum depended on the amount and size of the burrs obtained in the blade processing.

In the case of parts having internal surfaces, various methods of deburring are possible. The choice of the method depends on the results of cutting in terms of the number and size of burrs, as well as on the cost and utilization of work centers that use a suitable method of deburring.

The installation of steel and ceramic brushes and rubber-bonded abrasive wheels on the machine was tested. After processing with steel and ceramic brushes, burrs remained. When installing the rubber-bonded abrasive wheels on the machine, the result was not achieved; the wheels were destroyed under the influence of the lubricating-cooling technological medium. After applying the deburring by installing steel or ceramic brushes, the number of burrs was significantly reduced. The quality of the deburring with brushes installed on the machine was mostly affected by the root thickness, strength and the location of burrs.

The removal of burrs from the outer surfaces was carried out by tumbling, and the burrs were manually removed only from the inner surfaces. The plumbing operation involved cleaning with a scraper, drill, and abrasive wheels on a rubber bond. The complexity of the locksmith operation depended on the number, thickness of the root and the strength of the burrs.

Deburring in a thermal pulse unit is possible for parts made of beryllium bronze and the 29 NK alloy if the thickness of the burr root is not more than 0.1 mm and six times less than the minimum thickness of the part. Otherwise, thin-walled parts may be damaged. The size of the batch of parts loaded into the thermal pulse installation must be within the limits recommended for the selected processing mode [4]. When the batch size is reduced by 25% or more, ballast is loaded to change the volume of the chamber.

**Figure 2:**
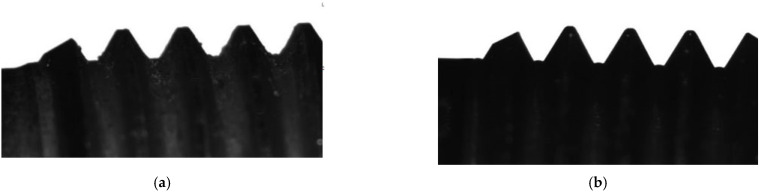
Thermal pulse screw thread processing: (**a**) the part having burrs before processing; (**b**) the part after processing in a rational mode of P = 1760 kPa; the burrs have been removed, and the surface quality and thread dimensions have been preserved.

Figure 2b shows the experimental results of removing burrs from the 29 NK alloy parts using the Pulsar VKF 3.250 thermal pulse unit. The initial treatment modes were as follows: the combustion temperature was T = 3500 °C, the pressure was P = 1800 kPa, the ratio of gas supply under pressure was P (C3H8) = 3.70 MPa and P (O2) = 6.65 MPa, the burning time was 20–30 milliseconds, and the mixing ratio of propane–oxygen was 1/2, being the stoichiometric parameters. When selecting the modes, the pressure was reduced to 1760 kPa. Before processing, the part had long, extending burrs that appeared during screw threading (Figure 2a), as well as small, long, extending and drop-shaped burrs on the inner surface. After the part was processed in a rational mode, the burrs were removed from the external and internal surfaces and the surface quality and dimensions of the part were preserved. Edge rounding did not occur in the rational mode. The overall dimensions of the part were the height of 10.2 mm, and the diameter of 5 mm. The results of the quality analysis of the thread surface showed that the surface roughness decreased. This happened due to the smoothing of the surface roughness by thermal-pulse treatment according to the unit manufacturer. In the case of the thin-walled parts, their own parameters for processing burrs were determined [21]. It was confirmed experimentally that the complete removal of burrs having a root thickness of 0.1 mm from thin-walled parts and threaded parts (more than 1/4–1/6 of the wall thickness) led to part geometry violation. In the case of parts having thin-walled surfaces, threads and other small-sized structural elements, whose geometry must be preserved, the rounding of the edges and chamfers required to be completed at the machining stage. Complete removal of the burrs without defect formation on the part occurred when the burr root thickness was within 1/4–1/6 of the minimum thickness of the part.

Deburring by means of deburring robots is only possible in the case of large volumes of workpieces. The cost of automating the deburring process is quite high and it is profitable only if there are two or three shifts of machines involved in producing such parts [4]. The efficiency of this deburring method does not depend on the quantity and parameters of the burrs.

An experiment performed on deburring by chemical etching showed that only thin burrs having a root thickness of up to 0.02 mm were removed, and burrs having a thick root were not removed, with the root remaining. The root thickness of the burrs obtained in the selected cutting conditions exceeded the thickness of 0.02 mm [4].

Burrs having a root size of up to 0.05 mm were removed with a scraper without forming “cuts”; larger burrs were removed by grinding for 0.5–1 min. It was established experimentally that the removal of burrs from the BrB2 beryllium bronze samples, processed in the modes of S = 6000 rpm, n = 0.03 mm/rev, f = 0.2 mm, was performed in 1.5 h–0.5 h, when grinding with a solution of the GA filler of 10 × 10. There was 0.5 h polishing in the presence of a solution of the OTEC PM-10 filler, and 0.5 h fine polishing in the case of a solution of the GA filler, 4 mm in diameter. The number of burrs on the samples varied from two to five pieces; the maximum root size was from 0.02 mm to 0.03 mm. The processing time for a sample with five burrs controlled manually was 15–20 s.

Removal of burrs from the BrB2 beryllium bronze samples processed in the modes of S = 8000 rpm, n = 0.05 mm/rev, and f = 0.3 mm was performed in 2.5 h. A total of 1 h was spent on grinding with a solution of the GA filler of 10 × 10. A total of 1 h was spent polishing with the OTEC PM-10 filler solution; a total of 0.5 h was used for fine polishing with the GA filler solution, to 4 mm in diameter. The number of burrs on the samples was from 9 to 13 pieces, and the maximum root size was from 0.07 mm to 0.08 mm. The processing time for a sample with 11 burrs controlled manually was 5 min.

As a result, we can conclude that the time for deburring by tumbling from the parts obtained in the selected modes was 1.67 times less than that in the modes at an increased feed and cutting speed, with manual deburring taking 15 times longer. The thermal impulse deburring of 1000 parts in the Pulsar VKF 3.250 took 20 min, and the deburring accompanied by subsequent manual deburring took 2 h and 5.5 person-hours. Minimizing the number and size of burrs reduces the cost of burr removal. The main burr parameter that limits the use of the thermal pulse deburring method is the root thickness. Setting the feed, speed and depth of the cut closer to the minimum values of the range recommended by the manufacturer ensures the planned wear resistance of the tool and the standard thickness of the burr root.

### 3.2. Experimental Study of the Influence of Blade Processing Modes of Small-Sized, High-Precision Parts Made of Beryllium Bronze and the 29 NK Alloy on the Parameters of Burrs

#### 3.2.1. Design of the Experiment

The choice of variable parameters and the determination of the level of variation were carried out on the basis of a preliminary study of the effect of cutting modes on the burr parameters. The surface of the lamella socket slot of a coaxial radio component made of beryllium bronze (Figure 1b) was used as a test surface. This surface is difficult to access for processing using deburring mechanical methods. The following parameters are accepted as variable parameters that affect the thickness of the burr root formed on small-sized parts made of beryllium bronze and the 29 NK alloy:

1. Feed S, mm/rev.
2. Number of spindle revolutions n, rpm.
3. Depth of cut f, mm.

The ranges of the cutting parameters recommended by the manufacturer for copper, brass, and bronze for turning operations are:

− cutting speed Vc is from 100 m/min to 500 m/min;
− feed S is from 0.01 mm/rev to 0.2 mm/rev; the cutting depth f is from 0.05 mm to 1.0 mm;
− feed S is from 0.05 mm/rev to 0.35 mm/rev; the cutting depth f is from 1 mm to 4 mm [21].

The manufacturers recommend the following ranges for cutting data for the 29 NK alloy in turning operations:

− cutting speed Vc is from 60 m/min to 120 m/min;
− feed S is from 0.01 mm/rev to 0.08 mm/rev; the depth of cut f is from 0.05 mm to 1 mm;
− feed S is from 0.05 mm/rev to 0.15 mm/rev; the cutting depth f is from 1 mm to 3 mm [21].

In this study, model 3 of 3-level factors was used, which represents a fractional factorial design for the experiment: for three factors (independent variables), having three levels of variation and the number of experiments n = 27; the number of repetitions was n = 3. The experiment was planned using the software product Statistica 6. The sequence of experiments was systematized according to the levels of variation. The stages of the experiment included coding factors, drawing up a plan for the matrix of the experiment, structuring the experiments, implementing the plan of the experiment, and checking the reproducibility of the experiments.

Table 5 and Table 6 show the natural and normalized levels of the factors used in the experimental plan.

#### 3.2.2. Definition of a Parametric Region with Rational Cutting Mode Parameters

As a result of a series of full-scale tests (processing of workpieces made of beryllium bronze and the 29 NK alloy in various combinations of cutting conditions), a working array was obtained from the output parameter: the maximum thickness of the burr root tg. Based on the results of the experiment, the variations and promising cutting conditions were determined, including the area of the worst combination of cutting conditions. A rational combination of parameters to ensure the quality of the process was determined using the Statistica 6 software (StatSoft Inc., Tulsa, OK, USA) package.

The results of the experiment on processing blanks made from beryllium bronze are described below.

**Figure 3:**
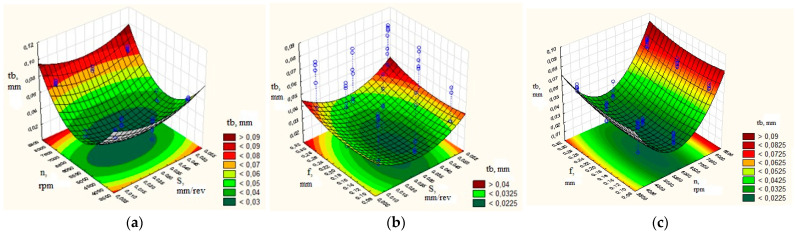
Determination of the parametric region with rational modes for workpieces made from beryllium bronze, with the effect on the thickness of the burr root: (**a**) feed and number of spindle revolutions; (**b**) depth of cut and feed; (**c**) number of spindle revolutions and the depth of cut.

To assess the influence of the turning mode parameters on the maximum thickness of the burr root, graphs of the response surface were plotted, as shown in Figure 3. The graphs (Figure 3) of the response surface were obtained using the Statistica 6 software package based on the experimental data.

The data in Figure 3a indicate that the smallest thickness of the burr root was achieved when the number of spindle revolutions was n = 6000 rpm. In this case, the cutting speed was 94.25 m/min—that is, the minimum speed recommended by the tool manufacturer, at a feed rate of 0.02 mm / rev, slightly higher than the recommended minimum. In the case of an increase as well as a decrease in the feed and according to the number of spindle revolutions, the quality of processing decreased (the thickness of the burr root increased).

Figure 3b shows the data obtained by setting the number of spindle revolutions at n = 6000 rpm. In this case, the smallest thickness of the burr root was obtained at a feed of S = 0.03 mm/rev and a depth of cut of 0.1 mm—that is, at the minimum feed recommended by the manufacturer, at a depth of cut in the recommended range. Increasing and decreasing the feed, and to a small extent the depth of cut, decreased the quality of processing (the thickness of the root of the burrs increased).

Figure 3c demonstrates that, by fixing the feed of S = 0.03 mm/rev, the smallest thickness of the burr root was obtained when spindle revolutions were n = 6000 rpm. The depth of cut was in the range from 0.1 mm to 0.3 mm—that is, the minimum cutting speed recommended by the manufacturer having a depth of cut in the recommended range. During increase and decrease in the number of spindle revolutions, the quality of processing decreased (the thickness of the root of the burrs increased).

**Figure 4:**
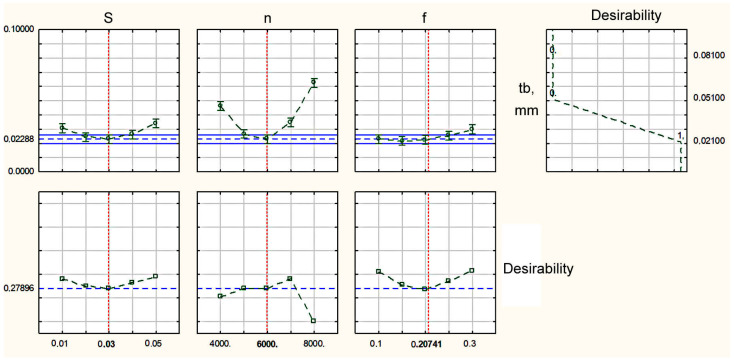
Profiles of predicted values and desirability functions of the values of processing parameters to obtain the minimum thickness of the burr root when turning workpieces made from beryllium bronze.

Analysis of the profiles of the predicted values and desirability functions displayed in Figure 4 shows that, in order to obtain the minimum thickness of the burr root, it was necessary to set S = 0.03 mm/rev and n = 6000 rpm, f = 0.20741 mm. In this case, the thickness of the burr root would be within 0.051 mm. The development of this mode until the tool was completely worn out showed that, in these modes, the planned wear resistance of the tool was ensured; the thickness of the burr root at a high degree of tool wear increased to 0.08 mm. This mode provided a normative thickness of the burr root within 0.088 mm in the case of the thermal pulse deburring of parts having thin-walled structural elements of a thickness of at least 0.36 mm.

**Figure 5:**
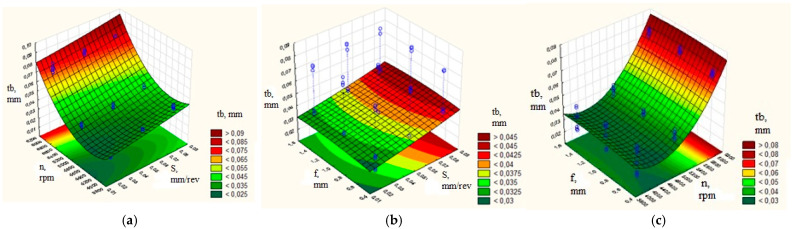
Determination of the parametric region having rational modes for workpieces made from alloy 29 NK, with the effect on the thickness of the burr root: (**a**) feed and number of spindle revolutions; (**b**) depth of cut and feed; (**c**) number of spindle revolutions and the depth of cut.

The results of the experiment on processing the workpieces made from the 29 NK alloy are described below. To assess the influence of the turning mode parameters on the maximum thickness of the burr root, graphs of the response surface were plotted, as shown in Figure 5.

The data in Figure 5a indicate that the thickness of the burr root was the smallest when the spindle revolutions were n = 4000 rpm, and when the cutting speed was 62.3 m/min; that is, it occurred at the minimum speed recommended by the tool manufacturer, and at a feed rate of 0.02 mm/rev that was slightly higher than the recommended minimum. When increasing the feed within the range recommended by the manufacturer, the size of the root of the burr increased slightly; the thickness of the root of the burr mostly increased owing to an increase in the number of spindle revolutions. In this case, the extremum was reached at low values of the number of spindle revolutions and feed.

Figure 5b shows the smallest thickness of the burr root at a feed of S = 0.02 mm/rev and a depth of cut of 0.5 mm; that is, at the minimum feed recommended by the manufacturer and at a depth of cut in the recommended range. Increasing by a slight degree of feed decreased the quality of processing (the thickness of the burr root increased).

Figure 5c shows that the smallest thickness of the burr root was obtained when the number of spindle revolutions was n = 4000 rpm and the cutting depth was in the range from 0.5 mm to 1.5 mm; that is, at the minimum cutting speed recommended by the manufacturer and at a cutting depth in the recommended range. When increasing the number of spindle revolutions, a decrease in the quality of processing occurred (the thickness of the burr root increased).

**Figure 6:**
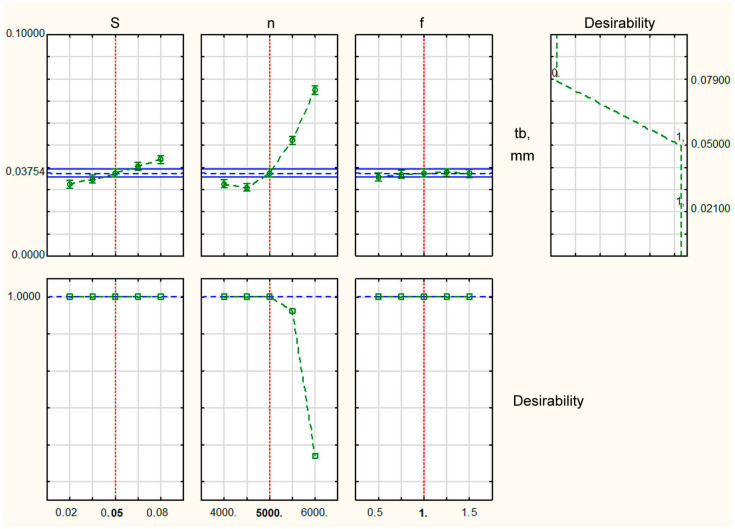
Profiles of predicted values and desirability functions of the values of processing parameters to obtain the minimum thickness of the burr root when turning workpieces made from alloy 29 NK.

Setting rational turning mode parameters, shown in Figure 3 and Figure 5 in dark green, resulted in an increase in the efficiency of the blade processing of small-sized parts made of beryllium bronze and the 29 NK alloy and enhanced the conditions for thermal pulse deburring. An analysis of the profiles of the predicted values and desirability functions displayed in Figure 6 shows that, in order to obtain the minimum thickness of the burr root, it was necessary to set the number of spindle revolutions from 4000 to 5000 rpm, and the feed and depth of cut in the range recommended by the manufacturer. In this case, the thickness of the burr root would be within 0.5 mm. The development of this mode until the tool was completely worn out showed that, in these modes, the planned wear resistance of the tool was ensured; the thickness of the burr root at a high degree of tool wear increased to 0.08 mm. This mode provided the standard thickness of the burr root within 0.088 mm for the thermal pulse deburring of parts having a structural element thickness of at least 0.36 mm.

#### 3.2.3. Construction of Empirical Regression Dependencies Allowing Description of the Effect of Blade Processing Modes of Small-Sized, High-Precision Parts Made of Beryllium Bronze and the 29 NK Alloy on the Parameters of Burrs

The construction of the regression dependence of the studied parameters was carried out in accordance with the method of a fractional factorial experiment. The implementation of this method was based on experiment design theory. We considered the choice of a regression model using the example of the parameter maximum thickness of the burr root when processing a workpiece made of beryllium bronze in accordance with the above experimental conditions. The factors used in the calculations were: ×1 (feed), ×2 (number of spindle revolutions), and ×3 (depth of cut). The results of the regression analysis are shown in Table 7. The table shows the regression coefficients for factors A—feed, B—number of spindle revolutions, and C—depth of cut. The significant factors of the mathematical model are shown in gray lines according to a p-criterion of less than the significance level.

Based on the results of the experiments, an incomplete square model of the dependence of the thickness of the burr root tb on the feed S, the number of spindle revolutions n, and the depth of cut f of the form was obtained:

(1) $$tb=0.3085-1.34017S-0.00009n+23.73563f^{2}$$

The coefficient of determination R^2^ = 0.92036; therefore, 92% of the variability of the deviation of the size of burrs occurring on workpieces made from beryllium bronze can be considered to have been due to the influence of the factors taken into account, and 8% was due to other factors.

A search for a regression model of the parameter maximum thickness of the burr root was carried out when machining a workpiece made from the 29 NK alloy. The results of the regression analysis are shown in Table 8.

Based on the results of the experiments, an incomplete linear model of the dependence of the burr root thickness tb of the number of spindle revolutions n was obtained in the form of:

(2) tb=0.324523−0.000141n

The coefficient of determination R^2^ = 0.97644; therefore, we can assume that 97% of the variability of the burr size deviation was due to the influence of the factors taken into account, and 3% was due to other factors.

The resulting polynomials enable establishing the parameters of the turning process to ensure manufacture of the products from beryllium bronze and the 29 NK alloy with a standard thickness of the burr root.

### 3.3. Technological Recommendations for Setting Rational Parameters for the Blade Processing of Small-Sized, High-Precision Parts Made of Beryllium Bronze and the 29 NK Alloy

To process small-sized, high-precision parts made of beryllium bronze and the 29 NK alloy, a precision cutting tool for micromechanics from the Swiss companies Applitec [23], Utilis [24], Fraisa [25], and IFANGER was used.

The use of CITIZEN high-precision CNC lathes with the recommended tool ensured high rigidity of the machine-tool-tool-part system. To reduce the temperature in the cutting area, oil cutting fluid was used.

The recommendations followed for setting the processing modes in the control program were as follows:

using equipment and tools for micromechanics, and lubricating and cooling the process medium;

in the case of beryllium bronze, the values of feed S and choice of the number of spindle revolutions n closer to the minimum value of the range recommended by the tool manufacturer to enable provision of the minimum thickness of the burr root to calculate the rational turning parameters, using a dependence of 1;

in the case of alloy 29 NK, the values of the number of spindle revolutions n were chosen to be closer to the average value of the range recommended by the tool manufacturer, providing the minimum thickness of the burr root to calculate rational turning parameters, using a dependence of 2;

the values can be increased to obtain the normalized thickness of the burr root (for the studied parts of no more than 0.088 mm) compared to the planned tool wear;

the processing strategy is that the burr should not reach the critical surface on the edges;

rationally selected modes are to be included in the directory of processing modes.

## 4. Discussion

This study is devoted to the development of recommendations for minimizing the size of burrs formed during the blade processing of small-sized, high-precision parts made of the BrB2 beryllium bronze and 29 NK alloy. An urgent scientific and technical problem has been solved to improve blade processing to ensure the conditions for subsequent physical and technical thermal pulse processing. This task is solved anew for each specific case concerning the dimension requirements of parts in manufacturing enterprises. General patterns can be identified concerning the influence of cutting modes on roughness. However, these data do not provide an exact answer to the question of the dimensions of burrs to be obtained and what their sizes will be. The results of the calculations based on these investigations for the studied alloys appear to be approximate. The results can inform manufacturers’ recommendations on modes. In each specific case (e.g., new dimensions of the workpiece, a new shape of the surface), a series of preliminary experiments require to be carried out to determine the optimal mode. These modes are not suitable for new sizes. The obtained results concerning the studied alloys (BrB2 with a confidence level of 92% and 29 NK with a confidence level of 97%) can significantly reduce the amount of preliminary experimental work required to obtain the desired modes.

A set of technical solutions has been proposed that makes it possible to increase the productivity of processing and to ensure high quality of the surfaces of parts after thermal impulse deburring.

**Figure 7:**
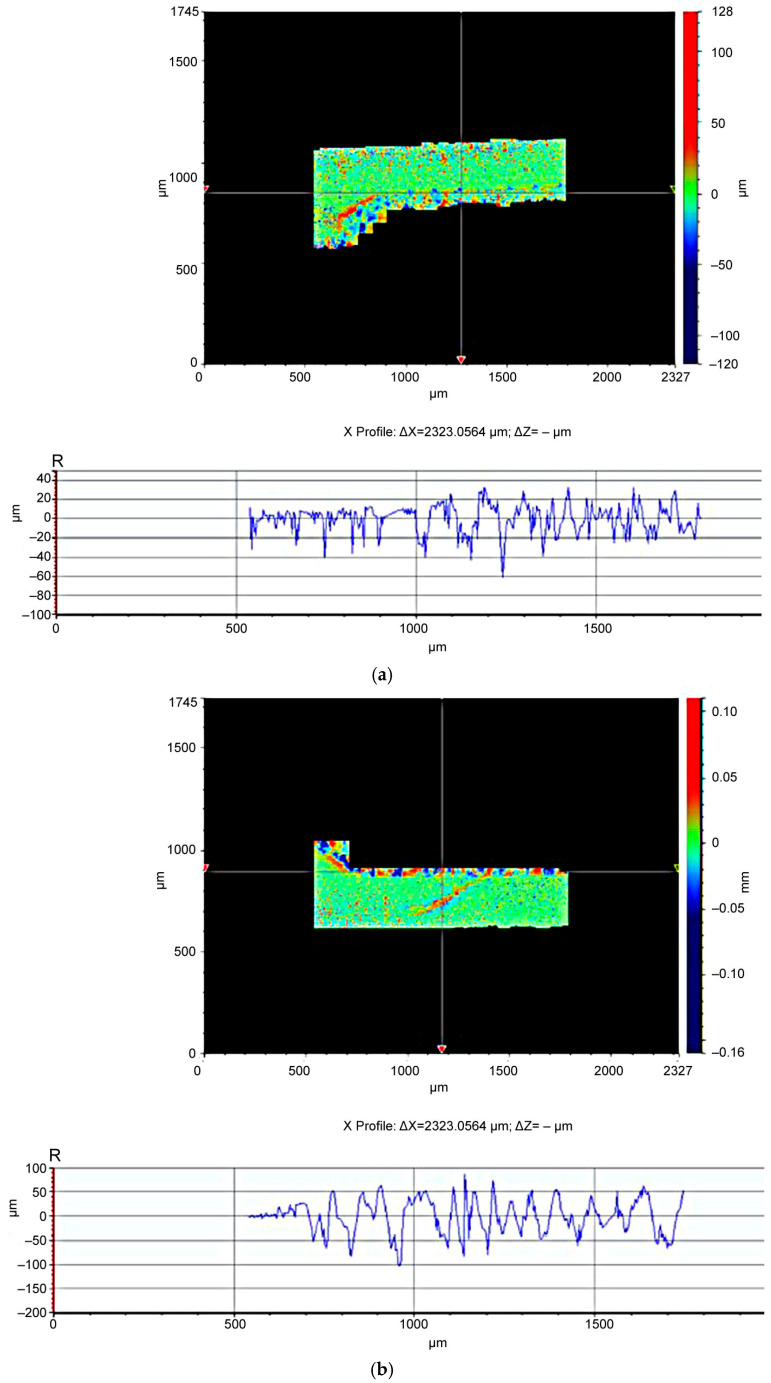
Surface quality of a beryllium bronze workpiece after turning: (**a**) having rational parameters; (**b**) when the feed and cutting speed increase.

1. It has been experimentally established that the thickness of the burr root remaining after turning workpieces made of the BrB2 beryllium bronze and 29 NK alloy depends on the parameters of the cutting mode during turning. For the BrB2 beryllium bronze, the thickness of the burr root is primarily affected by the value of the longitudinal feed during turning, by the cutting speed and by the depth of cut. For the 29 NK alloy, the thickness of the burr root depends only on the cutting speed. The difference in the dependencies is explained by the peculiarities of the physical and mechanical characteristics of these materials—that is, the Young’s modulus, strength and thermal conductivity [21]. Figure 7 shows the influence of the turning mode parameters on the quality of the machined surface of a beryllium bronze workpiece.
  The surface roughness of a beryllium bronze workpiece after processing at a feed rate of 0.03 mm/rev, a cutting speed of 100 m/min, and a cutting depth of 0.3 mm (Figure 7a) has the following characteristics:
  − an average distance between the highest peak and the lowest trough in the case of each sample length of Rz = 66.491 µm;
  − a maximum depth of the depression below the midline within one sample length of Rv = 60.598 µm; an average value of Rv over the evaluation length of 23.183 µm;
  − a maximum peak height above the midline within one sample length of Rp = 32.597 µm; an average Rp value over the evaluation length of 13.436 µm.
  The surface roughness of a beryllium bronze workpiece after processing at a feed rate of 0.05 mm/rev, a cutting speed of 125 m/min, and a cutting depth of 0.4 mm (Figure 7b) increases and amounts to:
  − an average distance between the highest peak and the lowest trough at each sample length of Rz = 141.377 µm;
  − a maximum depth of the depression below the midline within one sample length of Rv = 102.502 µm; an average value of Rv over the evaluation length of 35.892 µm;
  − a maximum height of the peak above the midline within one sample length of Rp = 87.756 µm; an average value of Rp over the evaluation length of 18.158 µm.
2. The rational parameters of the cutting mode were determined, providing rational conditions for subsequent physical and technical thermal pulse processing. The used equipment, the cutting tool, the cutting fluid, and the cutting conditions do not affect the thickness of the burr root in the same way. The following features are peculiar to the BrB2 beryllium bronze:
  − a minimum thickness of the burr root of less than 0.048–0.051 mm is provided, including a feed rate of 0.02–0.04 mm/rev, a cutting speed of 90–100 m/min, and a cutting depth of 0.2–0.3 mm;
  − the thickness of the burr root increases to 0.08–0.09 mm at a feed rate of 0.05–0.07 mm/rev, a cutting speed of 125–135 m/min, and a cutting depth of 0.4–0.6 mm.
  For the 29 NK alloy, the features are as follows:
  − a minimum thickness of the burr root of less than 0.04–0.06 mm is formed at a feed rate of 0.02–0.08 mm/rev, at a cutting depth of 0.5–1.5 mm, and at a cutting speed of 95–100 m /min;
  − an increase in the cutting speed to 110–120 m/min increases the root thickness to 0.08–0.09 mm.
  Figure 2 and Figure 4 show the change in the thickness of the burr root when the parameters of the turning mode are changed. One of the parameters is fixed, and changing the other two shows their effect on the thickness of the burr root. The range of the parameters within which the burr root thickness is minimal is highlighted in dark green. Figure 3 and Figure 5, produced using the Statistica 6 software package, show the values of the desirability function for obtaining the minimum burr roots.
3. The obtained empirical regression dependencies show the relationship between the thickness of the burr root and the parameters of the turning mode in a certain part of the range of values recommended by the tool manufacturer. In the case of the parts made of the BrB2 beryllium bronze, the dependencies set the thickness of the burr root at values of the longitudinal feed and the number of spindle revolutions (cutting speed) derived only from the first half of the range. When assigning increased values of the feed and the number of spindle revolutions (cutting speed), the thickness of the burr root increases significantly and exceeds the maximum allowable size. For the parts made of the 29 NK alloy, the dependencies set the thickness of the burr root at the values of the number of spindle revolutions (cutting speed) from the middle part of the range. When setting the number of spindle revolutions (cutting speed) closer to the maximum values of the range, the thickness of the burr root increases significantly and exceeds the maximum allowable size. Setting the cutting speed closer to the minimum values significantly increases the processing time of the machine.
4. The relationship between the completeness of physical-technical thermal pulse deburring and the dimensions of workpieces, namely, the thickness of their walls, has been established. The complete removal of burrs without causing defects in the part is ensured when the thickness of the burr root is within 1/4–1/6 of the minimum thickness of the part [21].

## 5. Conclusions

Minimizing the size and quantity of burrs that are formed on the surface of workpieces of small-sized, high-precision parts made of beryllium bronze and the 29 NK alloy during blade processing helps to reduce the cost of their removal and to ensure high quality processing.

The use of thermal pulse deburring is limited to the thickness of the burr root within 1/4–1/6 of the minimum thickness of the part.

The relationship between the parameters of the mode of turning workpieces and the size of the burrs formed on the machined surface of the workpiece has been established. It has been experimentally proved that an increase in the cutting speed and feed during turning has a more significant influence on increasing the burr root thickness in comparison with the depth of the cut.

The influence of the turning mode parameters and the blade processing conditions on the thickness of the burr root is scientifically substantiated, while the cutting mode parameters are assigned depending on the equipment used, the cutting tool and the lubricating-cooling technological medium.

The empirical regression dependencies have been obtained—they establish the relationship between the parameters of the cutting mode and the resulting thickness of the burr root. In the case of the BrB2 beryllium bronze, the dependence of the thickness of the burr root is described by a second-level polynomial equation. This enables specification of the rational range of feed, the number of spindle revolutions (cutting speed), and the quadratic dependence on the depth of cut. For the 29 NK alloy, the polynomial establishes a linear dependence of the burr root thickness on the number of spindle revolutions (cutting speed). The difference in dependencies of the polynomial equations is explained by the peculiarities of the physical and mechanical characteristics of these materials.

Based on the results of studying the influence of cutting modes on the burr parameters, recommendations were formulated for setting the cutting modes depending on the material of the workpiece.

## Figures and Tables

**Table 1:** Material properties of beryllium bronze [19].

Parameter	Value
Tensile strength	1110 MPa
Yield strength	1110 MPa
Young’s modulus	131 GPa
Brinell hardness	150 BHN
Melting point	866 °C
Thermal conductivity	115 W/mK
Heat capacity	420 J/g K

**Table 2:** Material properties of alloy 29 NK [17].

Parameter	Value
Tensile strength	518 MPa
Yield strength	276 MPa
Young’s modulus	207 GPa
Elongation percentage	30%
Thermal conductivity	17 W/mK
Thermal expansion coefficient	5.3 °C^−1^

**Table 3:** Chemical composition of beryllium bronze [19].

Element	Content (%)
Beryllium, Be	1.80–2.00
Lead, Pb	0.20–0.6
Silicon, Si	0.2
Aluminum, Al	0.2
Cobalt, Co	0.20 min
Copper, Cu	Balance

**Table 4:** Chemical composition of alloy 29 NK [20].

Element	Content (%)
Nickel, Ni	29
Cobalt, Co	17
Silicon, Si	0.2
Chromium, Cr	0.2
Carbon, C	0.02
Iron, Fe	Remainder

**Table 5:** Factors, intervals and levels of variation of the experiment plan for the blanks made from beryllium bronze.

Factor Level	S, mm/rev (x_1_)	n, rpm (x_2_)	f, mm (x_3_)
Upper (1)	0.01	4000	0.1
Main (0)	0.03	6000	0.2
Lower (−1)	0.05	8000	0.3
Variation interval	0.02	2000	0.1

**Table 6:** Factors, intervals and levels of variation of the experiment plan for the blanks made from 29 NK.

Factor Level	S, mm/rev (x_1_)	n, rpm (x_2_)	f, mm (x_3_)
Upper (1)	0.01	4000	0.1
Main (0)	0.03	6000	0.2
Lower (−1)	0.05	8000	0.3
Variation interval	0.02	2000	0.1

**Table 7:** Results of regression analysis of the maximum thickness of the burr root based on workpieces made from beryllium bronze of plan 3^(3)^. Regression: coefficient of determination R-sq.= 0.92036; adjusted 0.9139 (datasheet1) 3 3-level f, 1 blocks; residual sum of squared deviations SS = 0.0000294 RF maximum burr root thickness tb.

Factor	Regression Cofficients	Standard Error	Student’s *t*-Test	P-Test	−95% Confidence Interval	+95% Confidence Interval
Environments/custom member	0.30850	0.012136	25.4198	0.000000	0.28432	0.33269
A linear	−1.34017	0.195297	−6.8622	0.000000	−1.72930	−0.95103
A quadratic	0.38831	0.128009	3.0335	0.003333	0.13325	0.64338
B linear	−0.00009	0.000004	−23.4121	0.000000	−0.00010	−0.00008
B quadratic	0.00000	0.000000	24.5598	0.000000	0.00000	0.00000
C linear	−0.11981	0.052244	−2.2933	0.024675	−0.22391	−0.01571
C quadratic	23.73563	3.200225	7.4169	0.000000	17.35904	30.11222

**Table 8:** Results of regression analysis of the maximum thickness of the burr root on workpieces made from the 29 NK alloy of plan 3^(3)^. Regression: coefficient of determination R-sq.= 0. 97644; adjusted 0.9139 (Datasheet2) 3 3-level f, 1 blocks; residual sum of squared deviations SS = 0.00001 RF maximum burr root thickness tb.

Factor	Regression Coefficients	Standard Error	Student’s *t*-Test	P-Test	−95% Confidence Interval	+95% Confidence Interval
Environments/custom member	0.324523	0.018533	17.5103	0.000000	0.28759	0.361451
A linear	0.132922	0.084035	1.5817	0.117973	−0.03452	0.300365
A quadratic	0.534979	0.828021	0.6461	0.520217	−1.11489	2.184848
B linear	−0.000141	0.000007	−18.9487	0.000000	−0.00016	−0.000127
B quadratic	0.000000	0.000000	21.8181	0.000000	0.00000	0.000000
C linear	0.010074	0.006024	1.6725	0.098658	−0.00193	0.022076
C quadratic	−0.004296	0.002981	−1.4413	0.153722	−0.01024	0.001643

## Data Availability

The data presented in this study are available from the corresponding authors upon reasonable request.
